# The Infant Orphan Asylum, Wanstead

**Published:** 1904-12-31

**Authors:** 


### THE INFANT ORPHAN ASYLUM, WANSTEAD.

### DEFECTIVE ARRANGEMENT FOR SICKNESS.

Some months ago the Infant Orphan Asylum, Wanstead, brought itself into unpleasant prominence. Public attention and public curiosity were excited by a letter in the *Times*, signed by the consulting physician and consulting surgeon to the institution, announcing their resignation because of the omission on the part of the committee to provide proper isolation accommodation or nursing arrangements for the 500 orphans in their charge. A letter in which they placed their resignation before the committee was also published, in which it was pointed out that the arrangements for sickness and epidemic, recommended by the consulting staff some three years previously, and then approved by the committee, had now been set aside. This was done without further consultation with the medical staff, and their request that the matter might be discussed was not granted. The publication of these letters was followed by one from the treasurer to the governor, in which he stated that everything had been done which was necessary to provide for any probable sickness in the institution. To this Dr. Herringham, the consulting physician, replied by quoting the medical officer of health for Wanstead, who reported that the infirmary accommodation was inadequate, and the disinfection not to be relied upon. He further pointed out that the committee were incurring a great responsibility by putting their judgment on matters relating to hygiene before that of the medical officer they had themselves selected. Here the matter ended, and time probably has banished any public concern which was aroused. The committee of the Infant Orphan Asylum are pursuing the course they think best, and unless some very grave occurrence attracts public attention the matter will probably be forgotten. But the controversy has at any rate had one good effect. The London Orphan Asylum, recognising the danger of inadequately providing against epidemic, has taken advantage of the recommendations ignored by the Infant Orphan Asylum, and set its own house in order without delay. Possibly all the other well-managed orphan homes will, where it is necessary, do the same as speedily as possible.

But through this controversy round the affairs of the Infant Orphan Asylum, another and a wider issue forces itself upon our consideration. This affects the whole question of the care of orphan children in the present day. The increasing demands for improvements in education and hygiene are not to be met without a parallel increase in expenditure. Managers of institutions like the London Orphan Asylum may properly admit the force of public opinion and conform to it. Those who do not will sooner or later be held up to opprobrium. But when all has been done that science and humanity dictate to render the machinery of the orphanage above reproach, and as perfect for the purpose of maintaining and educating the orphan as is possible under such conditions, can we be satisfied that we are expending our money to the best advantage of the children we would care for by upholding the orphanage system? The thoughtful may well pause and study the many considerations which circle round the whole question.

